# Ten-Year Mortality after a Breast Cancer Diagnosis in Women with Severe Mental Illness: A Danish Population-Based Cohort Study

**DOI:** 10.1371/journal.pone.0158013

**Published:** 2016-07-27

**Authors:** Anette Riisgaard Ribe, Tinne Laurberg, Thomas Munk Laursen, Morten Charles, Peter Vedsted, Mogens Vestergaard

**Affiliations:** 1 Research Unit for General Practice, Department of Public Health, Aarhus University, Bartholins Allé 2, 8000 Aarhus C, Denmark; 2 Section for General Medical Practice, Department of Public Health, Aarhus University, Bartholins Allé 2, 8000 Aarhus C, Denmark; 3 Department of Experimental Clinical Oncology, Aarhus University Hospital, Nørrebrogade, 8000 Arhus C, Denmark; 4 National Centre for Register-Based Research, Department of Economics and Business Economics, Aarhus University, Fuglesangs Allé 4, 8210 Aarhus V, Denmark; National Institute of Health, ITALY

## Abstract

**Background:**

Breast cancer is the leading cause of cancer death in women worldwide. Nevertheless, it is unknown whether higher mortality after breast cancer contributes to the life-expectancy gap of 15 years in women with severe mental illness (SMI).

**Methods:**

We estimated all-cause mortality rate ratios (MRRs) of women with SMI, women with breast cancer and women with both disorders compared to women with neither disorder using data from nationwide registers in Denmark for 1980–2012.

**Results:**

The cohort included 2.7 million women, hereof 31,421 women with SMI (12,852 deaths), 104,342 with breast cancer (52,732 deaths), and 1,106 with SMI and breast cancer (656 deaths). Compared to women with neither disorder, the mortality was 118% higher for women with SMI (MRR: 2.18, 95% confidence interval (CI): 2.14–2.22), 144% higher for women with breast cancer (MRR: 2.44, 95% CI: 2.42–2.47) and 327% higher for women with SMI and breast cancer (MRR: 4.27, 95% CI: 3.98–4.57). Among women with both disorders, 15% of deaths could be attributed to interaction. In a sub-cohort of women with breast cancer, the ten-year all-cause-mortality was 59% higher after taking tumor stage into account (MRR: 1.59, 95% CI: 1.47–1.72) for women with versus without SMI.

**Conclusions:**

The mortality among women with SMI and breast cancer was markedly increased. More information is needed to determine which factors might explain this excess mortality, such as differences between women with and without SMI in access to diagnostics, provision of care for breast cancer or physical comorbidity, health-seeking-behavior, and adherence to treatment.

## Introduction

Women with severe mental illness (SMI), i.e., schizophrenia, schizoaffective disorders and bipolar affective disorders, have a two-fold higher risk of premature death compared to women without SMI [1, 2]. This corresponds to a life-expectancy gap of 15 years [3].

Although breast cancer is the leading cause of cancer death in women worldwide [4], it remains unclear whether the excess mortality in women with SMI can be partly explained by higher mortality after a breast cancer diagnosis. Mounting evidence suggests that comorbid physical conditions are generally under-diagnosed [5, 6, 7, 8] and under-treated [7, 9] in persons with SMI. The survival after a breast cancer diagnosis has improved during the last decades [10]. However, we do not know whether this tendency includes women with SMI, who have been shown to be less likely to receive mammography, to present with early stages of their breast cancer, and to receive appropriate treatment for breast cancer compared to those without SMI [11]. Lack of timely identification and appropriate treatment of breast cancer in women with SMI could lead to poorer prognosis after a breast cancer diagnosis.

Only five studies have evaluated breast-cancer-related mortality in women with SMI. One found a lower five-year relative survival after breast cancer for women with versus without schizophrenia (74% versus 79%) [12], and four studies found a 1.2- to 2.9-fold higher risk of breast-cancer-specific death in women with versus without SMI [13, 14, 15, 16]. However, as these studies are based on information from death certificates, the results may be biased; it is well-known that the validity of the stated causes of death is low [17], especially in persons with an underlying disease, such as SMI.

Using a large population-based cohort of all women in Denmark within a 32-year period, we aimed to estimate the all-cause mortality of women with SMI, women with breast cancer, and women with both SMI and breast cancer compared to women with neither disorder. Further, in a sub-cohort of women diagnosed with breast cancer, we aimed to estimate the ten-year all-cause mortality of women with SMI compared to women without SMI while taking into account the tumor stage.

## Methods

We conducted a population-based cohort study using information on Danish women from nationwide registers. All data were recorded with reference to the civil registration (CPR) number, a unique personal identification number assigned to all Danish residents. This number permits accurate linkage of recorded information at the individual level [18]. Until December 31, 1993, the diagnostic system used in the Danish registers was based on the International Classification of Diseases, 8th Revision (ICD-8). From January 1, 1994, the ICD-10 was used.

### Study population

Our study analyses were based on two cohorts: Our primary study cohort was established from the Danish Civil Registration System (DCRS) [18] and consisted of all women who were born in Denmark, at least 25 years of age and alive at some point during follow-up between 1980 and 2012. By linkage of the DCRS, the Danish Psychiatric Central Register (DPCR) [19] and the Danish Cancer Registry (DCR) [20], we divided the women in the cohort into four time-dependent disease categories (i.e., women with SMI and breast cancer, women with SMI only, women with breast cancer only, and a reference group comprising women with neither of the two disorders). Our secondary study cohort was established by restricting the primary cohort to women with a diagnosis of breast cancer only.

#### Severe mental illness

We identified all inpatient and outpatient contacts (i.e., psychiatric ambulatory care, excluding emergency contacts) to a psychiatric hospital for SMI between 1969 and 2012 from the DPCR (Appendix A in S1 File). The DPCR contains information on all admissions to psychiatric hospitals in Denmark since 1969 and all outpatient mental health contacts since 1995.

#### Breast cancer and tumor stage

We identified all breast cancer diagnoses between 1980 and 2012 from the DCR (Appendix B in S1 File). The DCR contains information on all cancers diagnosed in Denmark since 1943 and classified according to a modified version of the ICD-7 up to 1978 and hereafter according to the ICD-10 after conversion from the ICD-7 [20]. We excluded women with a breast cancer diagnosis before January 1, 1980 to ensure inclusion of incident cases only. We obtained information on tumor stage from the DCR using two definitions. Between 1980 and 2003, we used the register’s own definition of stage, i.e., “extent of disease”, which is categorized into localized, regional, distant (i.e., metastasis), or unknown [20]. Between 2004 and 2012, we translated tumor stage of breast cancers from the classification codes for tumor, node, and metastasis (TNM) into corresponding “extent of disease” by using the algorithm [21] shown in Appendix C in S1 File.

#### All-cause and cause-specific deaths

We obtained information on all-cause deaths between 1980 and 2012 from the DCRS, which includes information on gender, date of birth, vital status, and migration since 1968. We obtained information on deaths due to breast cancer between 1980 and 2010 from the Danish Register of Causes of Death (Appendix D in S1 File) [17], which contains information on causes of death and place of death of Danish residents from 1970 onwards.

#### Comorbid illnesses

We obtained information on diabetes between 1990 and 2012 from the Danish National Diabetes Register [22] using a validated algorithm (Appendix E in S1 File) [22, 23]. We obtained information on chronic diseases included in the Charlson Comorbidity Index (CCI) [24] between 1977 and 2012 from the Danish National Patient Register (DNPR) (Appendix F in S1 File) [25]. The CCI- score was calculated in a time-dependent manner by including inpatient and outpatient contacts and adding the individual weights of the diseases [24]. The DNPR contains information on all medical hospital contacts; inpatient contacts since 1977 and outpatient contacts since 1995. We obtained information on substance abuse (excluding tobacco abuse) between 1969 and 2012 from the DPCR or from the DNPR (Appendix G in S1 File).

### Statistical analysis

Follow-up started on January 1, 1980, and the women were censored on the day of death, on the day of emigration, or on January 1, 2012, whichever came first.

In our primary cohort of all women in Denmark, mortality rates (MRs) and mortality rate ratios (MRRs) for each disease category were calculated. As in our previous study [23], we calculated the attributable proportion due to interaction as a measure of the excess MRR for women with both conditions that was not explained by the independent effects of either condition [26].

In our secondary cohort of women with breast cancer, we calculated all-cause MRRs comparing women with and without SMI. We fitted four models of survival analysis to describe the ten-year all-cause mortality outcome and evaluated these for SMI, schizophrenia, and bipolar affective disorder, respectively. The first model included demographics (i.e., age and calendar period), the second model added tumor stage, the third model added medical comorbidity (i.e., diabetes and CCI score), and the fourth model added substance abuse. We performed four sub-analyses on our secondary cohort. First, we evaluated ten-year MRRs associated with SMI in subgroups according to demographics, tumor stage, medical comorbidity, and substance abuse. Second, we evaluated ten-year MRRs associated with SMI for four calendar periods (i.e., 1980–1987, 1988–1995, 1996–2003, and 2004–2011) and stratified for stage of breast cancer at diagnosis. Third, we evaluated MRRs associated with SMI for breast-cancer-specific death within ten years after the diagnosis of breast cancer. Finally, we evaluated conditional MRRs for the association between SMI and all-cause mortality for six different time periods after a breast cancer diagnosis (i.e., <1, 1–4, 5–9, 10–14, 15–19, and >20 years).

All MRRs were calculated using log-linear Poisson regression analysis with the logarithm of the person-years as an offset variable. We adjusted the MRRs for age and calendar period using two-year age- bands and time- bands. All variables, with the exception of tumor stage, were treated time-dependently. We used two-sided significance tests for all analyses; the level of statistical significance was set at P < 0.05. All analyses were performed with appropriate components of the STATA 13 (Stata Corporation, College Station, TX) statistical software program.

The study was approved by the Danish Data Protection Agency and the Danish Health and Medicine Authority. Ethical approval and informed consent were not needed, as all patient information was anonymized and de-identified prior to analysis by Statistics Denmark.

## Results

### All-cause mortality for women in Denmark

**Fig 1:**
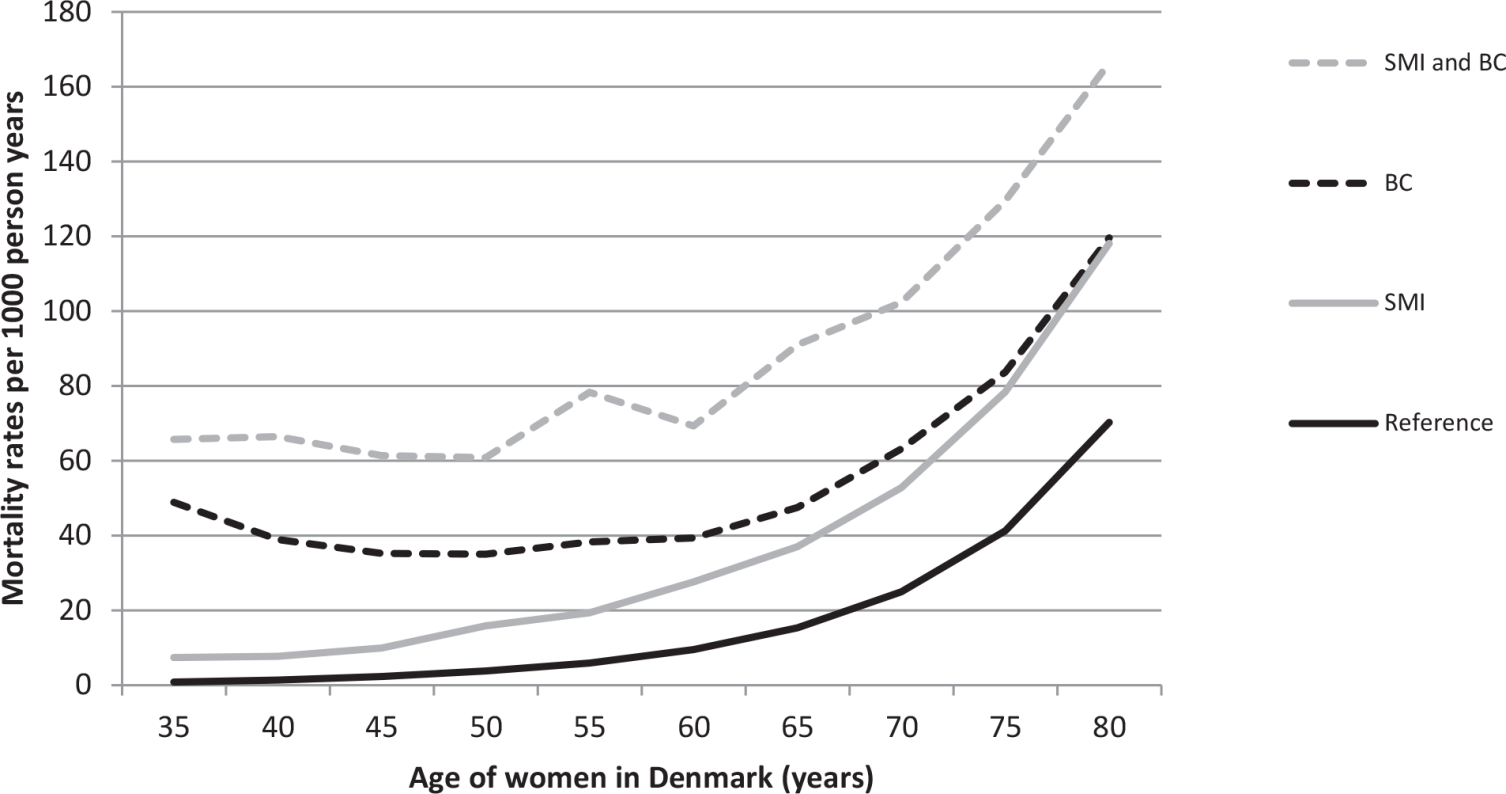
All-cause mortality rates (MRs) as a function of age for women with SMI only, women with breast cancer only, women with SMI and breast cancer, and women with neither of the two disorders (Denmark, 1980–2011). Abbreviations: MR: mortality rate; SMI: severe mental illness; BC: breast cancer; Reference: women from the general population with neither of the two disorders.

In our primary cohort, 2,665,963 women above 25 years of age were included; 864,190 of these died between 1980 and 2012. A total of 31,421 women had SMI (12,852 deaths), 104,342 had incident breast cancer (52,732 deaths), and 1,106 had both SMI and breast cancer (656 deaths). The MRs generally increased with increasing ages, whereas the curve for women with breast cancer was U-shaped (Fig 1). Compared to women with neither disorder, the MRR for women with SMI only was 2.18 (95% confidence interval (CI): 2.14–2.22), the MRR for women with breast cancer only was 2.44 (95% CI: 2.42–2.47), and the MRR for women with SMI and breast cancer was 4.27 (95% CI: 3.98–4.57) (Table 1). The combined effect of SMI and breast cancer on all-cause mortality was larger than the sum of effects of the two individual diseases (i.e., the attributable proportion due to interaction was 15.0% (95% CI: 9.0–20.9)) (Table 1).

**Table 1:** Mortality rate ratios (MRRs) for women with SMI, women with breast cancer and women with both SMI and breast cancer compared to women with neither SMI nor breast cancer in Denmark in 1980–2012 (N = 2,665,963).

MRR, neither SMI nor breast cancer	MRR, SMI (95% CI)	MRR, breast cancer (95% CI)	MRR, SMI and breast cancer (95% CI)	AP due to interaction, SMI and breast cancer (95% CI)
1 (ref)	2.18 (2.14–2.22)	2.44 (2.42–2.47)	4.27 (3.98–4.57)	15.0% (9.0–20.9)

Abbreviations: MRR: mortality rate ratio; SMI: severe mental illness; CI: confidence interval. The MRRs were adjusted for age and calendar period

### Ten-year all-cause mortality after a breast cancer diagnosis

**Fig 2:**
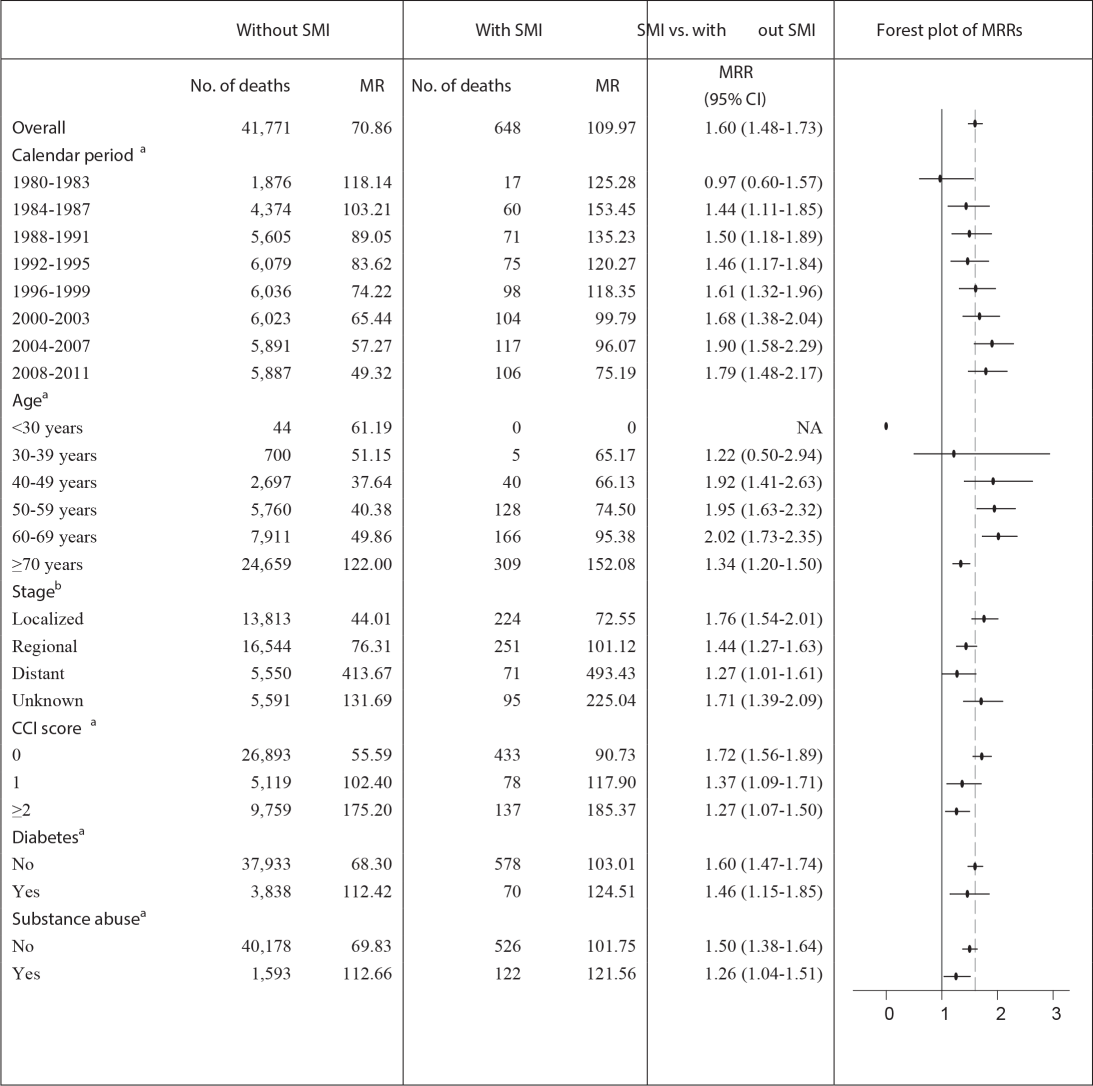
Ten-year all-cause MRRs for women with SMI and breast cancer compared to women with breast cancer only. Abbreviations: MRR: mortality rate ratio; MR: mortality rate; CI: confidence interval; CCI score: Charlson Comorbidity Index score (excluding cancers). The MRRs were adjusted for age and calendar period. ^a^Each death is assigned to the category at the time of death (for example, the number of deaths are counted for each calendar period category at the time of death: if a person died in 1981, this death will be assigned to the calendar period category 1980–1983). ^b^Number of deaths are counted for each tumor stage category at the time of diagnosis.

In our secondary cohort, 105,448 women with incident breast cancer were included, hereof 1,106 women with SMI (Table 2). In total, 648 (58.6%) women with SMI and 41,771 (40.0%) women without SMI died during up to ten years of follow-up (Fig 2).

**Table 2:** Number of breast cancer cases (N (%)) and their characteristics at time of diagnosis, 1980–2012 (N = 105,448).

	Without SMI	With SMI
**Total number of breast cancer cases**	104,342 (100%)	1,106 (100%)
**Calendar period**		
1980–1983	9,408 (9.0%)	73 (6.6%)
1984–1987	10,176 (9.8%)	87 (7.9%)
1988–1991	11,128 (10.7%)	102 (9.2%)
1992–1995	12,235 (11.7%)	113 (10.2%)
1996–1999	13,248 (12.7%)	146 (13.2%)
2000–2003	14,567 (14.0%)	166 (15.0%)
2004–2007	14,995 (14.4%)	184 (16.6%)
2008–2011	18,585 (17.7%)	235 (21.3%)
**Age at breast cancer diagnosis**		
<30 years	357 (0.3%)	1 (0.1%)
30–39 years	4,307 (4.1%)	21 (1.9%)
40–49 years	15,706 (15.1%)	156 (14.1%)
50–59 years	23,919 (22.9%)	304 (27.5%)
60–69 years	26,960 (25.8%)	309 (27.9%)
≥70 years	33,093 (31.7%)	315 (28.5%)
**Stage of breast cancer**		
Localized	48,763 (46.8%)	458 (41.5%)
Regional	38,837 (37.3%)	434 (39.2%)
Distant	6,473 (6.2%)	75 (6.8%)
Unknown	9,300 (8.9%)	124 (11.2%)
Missing	806 (0.8%)	14 (1.3%)
**CCI score**^**a**^		
0	87,651 (84.0%)	895 (80.9%)
1	7,936 (7.6%)	92 (8.3%)
≥2	8,755 (8.4%)	119 (10.8%)
**Diabetes**		
No	99,234 (95.1%)	1,012 (91.5%)
Yes	5,108 (4.9%)	94 (8.5%)
**Substance abuse**		
No	101,856 (97.6%)	919 (83.1%)
Yes	2,486 (2.4%)	187 (16.9%)

^a^Charlson Comorbidity Index (CCI) score included all of the 19 chronic diseases included in the CCI, except for cancer-related diseases. The distribution of the four most prevalent diseases from the CCI (with SMI vs. without SMI): cerebrovascular disease (4.5% vs. 4.0%), chronic pulmonary disease (3.9% vs. 3.2%), ulcer disease (4.2% vs. 2.3%), and connective tissue disease (1.5% vs. 2.1%).

Women with SMI had a 60% higher risk of death within ten years after a breast cancer diagnosis (MRR: 1.60, 95% CI: 1.48–1.73) compared to women without SMI (Table 3A). This risk was unaffected when adjusted for tumor stage (MRR: 1.59, 95% CI: 1.47–1.72), but attenuated when adjusted for medical comorbidities and substance abuse (MRR: 1.46, 95% CI: 1.36–1.58) (Table 3A). The MRR was lower for women with bipolar affective disorder (MRR: 1.39, 95% CI: 1.22–1.59) than for women with schizophrenia (MRR: 1.73, 95% CI: 1.57–1.90) (Table 3A). The ten-year MRR decreased by increasing stage of breast cancer and increasing CCI- score, but it increased by increasing calendar period (Fig 2). When plotted as a function of calendar period, the ten-year MRRs tended to increase over time for women with a localized or regional breast cancer stage, but not for distant stages, although not statistically significantly (S1 Fig).

**Table 3:** All-cause mortality and breast-cancer-specific mortality.

**A: Mortality rate ratios (MRRs) for the all-cause mortality ten years after a breast cancer diagnosis for women with SMI and breast cancer compared to women with breast cancer only (N = 42,419), 1980–2012**						
	**Total deaths**		**MRR (95% CI)**			
	**Without SMI**	**With SMI**	**Model 1**^a^	**Model 2** ^b^	**Model 3** ^c^	**Model 4**^**d**^
With SMI vs. without SMI	41,771	648	1.60 (1.48–1.73)	1.59 (1.47–1.72)	1.57 (1.45–1.70)	1.46 (1.36–1.58)
With schizo vs. without schizo^†^	41,998	421	1.73 (1.57–1.90)	1.70 (1.54–1.87)	1.71 (1.56–1.89)	1.60 (1.45–1.76)
With bipol vs. without bipol^††^	42,192	227	1.39 (1.22–1.59)	1.41 (1.24–1.61)	1.35 (1.18–1.54)	1.26 (1.10–1.43)
**B: MRRs for breast-cancer-specific cause of death ten years after a breast cancer diagnosis for women with SMI and breast cancer compared to women with breast cancer only (N = 26,078), 1980–2010**						
	**Total deaths**		**MRR (95% CI)**			
	**Without SMI**	**With SMI**	**Model 1**^a^	**Model 2** ^b^	**Model 3** ^c^	**Model 4**^**d**^
With SMI vs. without SMI	25,731	347	1.38 (1.24–1.54)	1.35 (1.21–1.50)	1.34 (1.21–1.49)	1.29 (1.16–1.44)
With schizo vs. without schizo^†^	25,840	238	1.55 (1.36–1.76)	1.49 (1.31–1.70)	1.49 (1.31–1.70)	1.44 (1.27–1.64)
With bipol vs. without bipol^††^	25,969	109	1.12 (0.93–1.35)	1.11 (0.92–1.34)	1.09 (0.90–1.32)	1.05 (0.87–1.27)

Abbreviations: MRR: mortality rate ratio; SMI: severe mental illness; CI: confidence interval; schizo: schizophrenia; bipol: bipolar affective disorder.

^a^Adjusted for age and calendar period.

^b^Also adjusted for tumor stage of breast cancer.

^c^Also adjusted for medical comorbidity (i.e., diabetes and the CCI -score, excluding diabetes-related diseases and cancers).

^d^Also adjusted for substance abuse.

^†^The ‘No schizophrenia’ -group comprised persons in the general population, including persons with bipolar affective disorder.

^††^The ‘No bipolar affective disorder’ -group comprised persons in the general population, including persons with schizophrenia.

### Breast-cancer-specific death ten years after a breast cancer diagnosis

In our secondary cohort, 347 (58.0%) of the deaths in women with breast cancer and SMI and 25,731 (66.3%) of the deaths in women with breast cancer but no SMI were classified as caused by breast cancer within ten years after a breast cancer diagnosis (Table 3B). The ten-year breast-cancer-specific MRR was 38% higher (MRR: 1.38, 95% CI: 1.24–1.54) for women with versus without SMI, and 55% higher for women with versus without schizophrenia (MRR: 1.55, 95% CI: 1.36–1.76), but the MRR was not statistically higher for women with versus without bipolar affective disorder (MRR: 1.12, 95% CI: 0.93–1.35) (Table 3B).

### All-cause mortality for different time periods after a breast cancer diagnosis

In our secondary cohort, the conditional MRRs for the association between SMI and all-cause mortality were relatively stable when evaluated for different time periods within 32 years of follow-up after a breast cancer diagnosis (S2 Fig).

## Discussion

This large nationwide cohort study of all women in Denmark showed that the all-cause mortality was 118% higher among those with SMI only, 144% higher among those with breast cancer only, and 327% higher among those with both SMI and breast cancer compared to women with neither of the two disorders. Further, the combined effect of SMI and breast cancer on all-cause mortality was larger than the sum of effects of the two individual diseases, as interaction between SMI and breast cancer accounted for 15% of all deaths. Among women diagnosed with breast cancer, the ten-year mortality after breast cancer diagnosis was 60% higher for all-cause death and 38% higher for breast-cancer-specific death for women with versus without SMI. This association was not entirely explained by tumor stage or comorbidity.

Several circumstances may explain why women with SMI have a higher mortality after a breast cancer diagnosis. Women with SMI and breast cancer may have delayed detection of their breast cancer due to suboptimal health-seeking behavior and health-care-utilization, such as lower awareness of adverse symptoms, not performing self-examination of breasts, and not participating in screening [11]. This hypothesis is supported by studies showing that women with SMI and breast cancer present with more advanced cancer disease at the time of diagnosis [11]. In our study, women with SMI were less likely to present with a localized breast cancer. Adjusting for tumor stage did not change the results much, which suggests that tumor stage might not play a major role for the association between SMI and mortality after breast cancer diagnosis. Furthermore, women with SMI and breast cancer could experience disparities in the provision of care for breast cancer [11] or for other comorbid physical conditions [7, 9]. Women with SMI may display reduced likelihood of accepting appropriate care if a breast mass is noticed [11]. Further, some physicians might be reticent about treating these patients according to clinical guidelines due to appropriate concern that women with SMI might have difficulties managing potential side effects of the treatment at home owing to lack of effective coping strategies or limited social network. Unfortunately, information on clinically important factors such as treatment modalities for breast cancer (i.e., type of surgery and adjuvant therapies), treatment of comorbidities, and adherence patterns was unavailable and therefore not included in the analyses.

Persons with SMI often have comorbid physical conditions [27, 28] and substance abuse [8]. Comorbidity in breast cancer patients is associated with poorer overall survival [29] and reduced likelihood of receiving surgery and adjuvant therapy, such as chemotherapy, radiation therapy, and tamoxifen treatment [29]. However, it remains unclear to what extent this suboptimal treatment reflects physician concern about toxicity due to comorbid conditions, low quality of medical health care, patient discontinuation of treatment caused by intolerable side effects, or poor adherence [29]. Nevertheless, limitations in treatment modalities can adversely affect the survival in women with breast cancer and comorbid conditions [30]. Furthermore, poor health status in women with breast cancer may interact with the breast malignancy and increase both the risk of death from breast cancer and from other causes [30]. As our primary outcome of interest was all-cause death, we did not only study the effect of breast cancer on survival; we also studied the contribution of comorbidity and death from other causes than breast cancer. Nevertheless, our outcome estimate did not change substantially when we adjusted for comorbidity. However, physical comorbidity is known to be under-diagnosed in persons with SMI [5, 6, 7, 8, 28], and further, less severe cases of comorbidity that did not lead to hospitalizations were not included in our comorbidity measure. Therefore, the significance of comorbidity could be underestimated in our study.

We found that the MRR tended to be higher among women with localized breast cancer than among women with breast cancer and metastasis. This is not surprising as the survival in women with advanced breast cancer is poor, and the additional effect of SMI on mortality on a relative scale is small. Still, among women with potentially curable breast cancer (i.e., localized breast cancer), the higher MRR may be explained by disparities in the health care provision or health care utilization for women with SMI. However, differential classification of tumor stage in women with and without SMI cannot be ruled out as the proportion of localized stages was slightly lower and the proportion of missing and unknown stages was slightly higher among women with SMI.

The survival after a breast cancer diagnosis has improved during the last decades, presumably due to enhanced treatment methods, early intensive treatment, and mass screening [10]. However, it is unknown whether women with SMI have profited from these advances in health care provision. We found that the ten-year MRRs associated with SMI tended to increase over calendar time for localized and regional stages, but not for distant stage, which might indicate that women with SMI may not have equally benefitted from improvements in treatment of early stage breast cancers. However, the differences were small, did not reach statistical significance, and may be due to changes in the composition of the SMI cohort over time that were not accounted for by our adjustments. Therefore, these results should be interpreted cautiously and confirmed in other studies before any inferences can be drawn. Still, these findings are consistent with studies which found evidence for a widening mortality gap over time between persons with and without SMI [1, 31].

Four previous studies found a 1.2- to 2.9-fold higher risk of breast-cancer-specific death in women with SMI compared to without SMI [13, 14, 15, 16]. The inconsistency of the estimates may, at least partly, be due to low sample size and statistical imprecision. Further, studies based on information from death certificates may be biased due to low validity of the causes of death [17] and cause-of-death attribution bias [32] (i.e., the cause-of-death registration may be biased by the physician’s knowledge about underlying diseases in the deceased). In addition, one study found a five-year relative survival after breast cancer of 74% in women with schizophrenia and 79% in women without schizophrenia [12].

Our study has several strengths, including the large population-based cohort and complete follow-up and registration of breast cancer cases. Therefore, it is unlikely that bias due to selection of study participants or loss to follow-up can explain our findings. The validity of the key variables is known to be high; the positive predictive value for a diagnosis of schizophrenia is 87% [33], the correctness for a diagnosis of breast cancer is 99% [34], and the completeness of all-cause death is close to 100% [18]. Thus, information bias is an unlikely explanation for our results. Most importantly, we explored the contribution of SMI, breast cancer, and the interaction between the two disorders on the mortality of all women in Denmark. In addition, we explored the significance of SMI on the mortality after a breast cancer diagnosis in a restricted cohort of women with breast cancer. The combination of these findings suggested that not only was the mortality for women with SMI considerably higher after breast cancer of similar tumor stage compared to women without SMI; this excess mortality could not be accounted for by a higher mortality of women with SMI in general, as the combined effect of SMI and breast cancer on all-cause mortality was larger than the sum of effects of the two individual diseases.

However, our study also has some limitations. Our breast cancer cohort comprised women who had been diagnosed with breast cancer and the health care contact already established. Thus, women with SMI and breast cancer may comprise fairly healthy women as the most severely affected women with SMI might not even be diagnosed with their breast cancer or surviving until the age of cancer diagnosis [3]. Our results might, therefore, not be generalizable to all women with SMI, but they may, nevertheless, represent conservative estimates of the mortality after breast cancer among women with SMI in general. The validity of tumor stage classification of breast cancer varies; around 90% of localized or regional disease is correctly staged in the DCR, whereas this is only the case for around 65% of breast cancers with metastasis [34]. Thus, the analyses including metastatic stage should be interpreted with caution. In addition, the registration of cause of death in women with SMI in our study could also be subject to differential misclassification caused by expected higher rates of death from external causes, cardiovascular disease, diabetes, or infections in persons with SMI [2, 23, 35]. Further, some important clinical information, such as life style factors (e.g., smoking, physical inactivity, and high BMI) generally associated with SMI [8, 36] and poorer prognosis after breast cancer [30, 37, 38], was not included in our analyses due to unavailability in the registers. Therefore, residual confounding cannot be excluded.

More studies are needed to determine which factors could explain the excess mortality demonstrated in our study, such as differences between women with and without SMI in access to diagnostics and screening, provision of care for breast cancer or physical comorbidity, health-seeking behavior, or adherence to treatment recommendations. Once such factors have been identified, it calls for studies exploring the significance of a collaborative care approach [11], which has proven effective in the treatment of depression comorbid with cancers [39]. Several efficient treatment options for breast cancer exist, which greatly enhance the survival in breast cancer patients. However, treatment of breast cancer in women with SMI still poses a clinical challenge [11]. Nevertheless, identifying increased mortality in women with both SMI and breast cancer is the first step to develop tailored interventions aimed at reducing the excess mortality in this group of patients.

## Conclusions

The combined effect of SMI and breast cancer on all-cause mortality was larger than the sum of effects of the two individual diseases. Further, the mortality after a breast cancer diagnosis was markedly increased in women with versus without SMI, and this association was not entirely explained by tumor stage or comorbidity. Our findings indicate that increased mortality after breast cancer could partly explain why women with SMI experience a life-expectancy gap of 15 years. More studies are needed to determine which factors could explain this excess mortality, such as differences between women with and without SMI in access to diagnostics and screening, provision of care for breast cancer or physical comorbidity, health-seeking behavior, or adherence to treatment recommendations.

## Supporting Information

S1 Fig Ten-year all-cause MRRs for women with SMI and breast cancer compared to women with breast cancer only as a function of calendar period stratified for tumor stage at diagnosis. Abbreviations: MRR: mortality rate ratios; SMI: severe mental illness; localized: localized tumor stage of breast cancer; regional: regional tumor stage of breast cancer; distant: breast cancer with metastasis. All MRRs were adjusted for age.
(EPS)

S2 Fig Conditional MRRs for all-cause mortality for different periods of time after a breast cancer diagnosis for women with SMI and breast cancer compared to women with breast cancer only. Abbreviations: MRR: mortality rate ratio, SMI: severe mental illness. All MRRs were adjusted for age and calendar period.
(EPS)

S1 File Appendices. (PDF)
