# Supplementary material for: Ten-Year Mortality after a Breast Cancer Diagnosis in Women with Severe Mental Illness: A Danish Population-Based Cohort Study
Source: PLoS One. 2016 Jul 27;11(7):e0158013. doi: 10.1371/journal.pone.0158013 (PMC4963132; doi:10.1371/journal.pone.0158013)
Supplement: S1 File — (PDF) [file pone.0158013.s003.pdf]

**Appendix A: Information on severe mental illness (SMI)[a] obtained from the Danish Psychiatric Central Register**		
	**ICD-8**	**ICD-10**
Schizophrenia and schizoaffective disorders	295, 296.8	F20, F25
Bipolar affective disorders	296.19, 296.39	F30, F31

[a]Persons were classified with SMI at the date of the first contact for one of the diagnoses schizophrenia, bipolar affective disorder, or schizoaffective disorder. Persons with a diagnosis of schizoaffective disorder were categorized as schizophrenia. A diagnosis of schizophrenia overruled a diagnosis of bipolar affective disorder.

**Appendix B: Information on breast cancer obtained from the Danish Cancer Registry**	
	**ICD-10**
Breast cancer	C50.0-50.9

**Appendix C: Information on tumor stage obtained from the Danish Cancer Registry and classified according to the tumor, node and metastasis (TNM) classification system**			
	**Tumor**	**Node**	**Metastasis**
Localized	T1-4	N0	M0
	T1-2	N0	Mx
	T1	Nx	M0x
Regional	T1-4,x	N1-3	M0
Distant	Any T	Any N	M1
Unknown	T2-4,x	Nx	M0-x
	T3-4,x	N0	Mx
	T1-4,x	N1-3	Mx
	T0	N1-3	M0-1x
	T0	N0,x	M1

**Appendix D: Information on breast cancer deaths in death certificates obtained from the Danish Register of Causes of Death**		
	**ICD-8**	**ICD-10**
Breast cancer	174.00-174.99	C50.0-C50.9

Appendix E: Information on diabetes obtained from the Danish National Diabetes Register

Algorithm: Individuals were classified as having diabetes on the date when at least one of the following six criteria was met:

1. A diagnosis of diabetes made at any Danish hospital registered in the Danish National Patient Register (ICD-8:249, 250; ICD-10:E10-14, H36.0, O24, excluding O24.4).
2. A referral to chiropody of diabetic patients as registered in the Danish National Health Service Register.
3. Five blood glucose measurements within one year registered in the Danish National Health Service Register.
4. Two blood glucose measurements per year for five consecutive years registered in the Danish National Health Service Register.
5. Two redeemed prescriptions for oral anti-diabetic drugs within six months registered in the Danish National Prescription Registry.
6. Two redeemed prescriptions for insulin registered in the Danish National Prescription Registry.

Appendix F: Information on the Charlson Comorbidity Index (CCI) obtained from the Danish National Patient Register

	ICD-8	ICD-10
Myocardial infarction	410	I21-I23
Congestive heart failure	427.09, 427.10, 427.11, 427.19, 428.99, 782.49	I50, I11.0, I13.0, I13.2
Peripheral vascular disease	440-445	I70-I74, I77
Cerebrovascular disease	430-438	I60-I69, G45, G46
Dementia	290.09-290.19, 293.09	F00-F03, F05.1, G30
Chronic pulmonary disease	490-493, 515-518	J40-J47, J60-J67, J68.4, J70.1, J70.3, J84.1, J92.0, J96.1, J98.2, J98.3
Connective tissue disease	712, 716, 734, 446, 135.99	M05, M06, M08, M09, M30-M36, D86
Ulcer disease	530.91, 530.98, 531-534	K22.1, K25-K28
Mild liver disease	571, 573.01, 573.04	B18, K70.0-K70.3, K70.9, K71, K73, K74, K76.0
Diabetes mellitus	249.00, 249.06, 249.07, 249.09, 250.00, 250.06, 250.07, 250.09	E10.0, E10.1, E10.9, E11.0, E11.1, E11.9
Hemiplegia	344	G81, G82
Moderate/severe renal disease	403,404,580-584, 590.09, 593.19, 753.10-753.19, 792	I12, I13, N00-N05, N07, N11, N14, N17-N19, Q61
Diabetes mellitus with chronic complications	249.01-249.05, 249.08, 250.01-250.05, 250.08	E10.2-E10.8, E11.2-E11.8
Any tumor	140-194	C00-C75
Leukemia	204-207	C91-C95
Lymphoma	200-203, 275.59	C81-C85, C88, C90, C96
Moderate/severe liver disease	070.00, 070.02, 070.04, 070.06, 070.08, 573.00, 456.00-456.09	B15.0, B16.0, B16.2, B19.0, K70.4, K72, K76.6, I85
Metastatic solid tumor	195-198, 199	C76-C80
AIDS	079.83	B21-B24

Appendix G: Information on substance abuse obtained from the Danish National Patient Register and the Danish Psychiatric Central Research Register

	ICD-8	ICD-10
**Drug-related abuse**		
Opioids	304.09, 304.19	F11.0–F11.9
Cannabinoids	304.59	F12.0–F12.9
Sedatives/hypnotics	304.29, 304.39	F13.0–F13.9
Cocaine	304.49	F14.0–F14.9
Other stimulants	304.69	F15.0–15.9
Hallucinogens	304.79	F16.0–F16.9
Other and multiple drugs	304.89, 304.99	F18.0–F19.9
**Alcohol-related abuse**		
Alcohol psychosis and abuse syndrome	291.09–291.99, 303.09-303.99	F10.0–F10.9
Cirrhosis and steatosis of the liver	571.09, 571.10, 571.19	K70.0–K70.9
Esophageal varices	456.00, 456.01, 456.09	I85.0–I85.9
